# Supplementary material for: Changes in the lipidome in type 1 diabetes following low carbohydrate diet: Post‐hoc analysis of a randomized crossover trial
Source: Endocrinol Diabetes Metab. 2021 Jan 4;4(2):e00213. doi: 10.1002/edm2.213 (PMC8029500; doi:10.1002/edm2.213)
Supplement: Supplementary file 1 — Supplementary Material [file EDM2-4-e00213-s001.docx]

# Supplementary Material

# Changes in the lipidome in type 1 diabetes following low carbohydrate diet: post-hoc analysis of a randomized crossover trial

Naba Al-Sari, Signe Schmidt, Tommi Suvitaival, Min Kim, Kajetan Trost, Ajenthen G. Ranjan, Merete B. Christensen, Anne J. Overgaard, Flemming Pociot, Kirsten Nørgaard, Cristina Legido-Quigley

This document contains the following Supplementary Materials:

- Supplementary Methods
- Supplementary Figures
- Supplementary Tables

# **Supplementary Methods**

**Supplementary Methods 1 internal standards used in the lipid extraction method.**

Following nine different internal standards were used; 1,2-dimyristoyl-sn-glycero-3-phospho(choline-d13) (PC(14:0)-d13)), 1,2,3-triheptadeca­noylglyc­erol (TG(17:0/17:0/17:0)) and 3β-hydroxy-5-cholestene 3-linoleate (ChoE(18:2)). 1,2-diheptadecanoyl-sn-glycero-3-phosphoethan­olamine (PE(17:0/17:0)), N-heptadecanoyl-D-erythro-sphingosylphosphorylcholine (SM(d18:1/17:0)),N-hepta­decanoyl-D-erythro-sphingo­sine (Cer(1/17:0)-d18), 1,2-diheptadecanoyl-sn-glycero-3-phosphocholine (PC(17:0/17:0)), 1-heptadecanoyl-2-hydroxy-sn-glycero-3-phospho­choline (LPC(17:0)), 1-palmitoyl-d31-2-oleoyl-sn-glycero-3-phosphocholine (PC(16:0/d31/18:1)), 1-hexadecyl-2-(9Z-octadecenoyl)-sn-glycero-3-phos­phocho­line (PC(16:0e/18:1(9Z))), 1-(1Z-octadecenyl)-2-(9Z-octadecenoyl)-sn-glycero-3-phos­phocholine (PC(18:0p/18:1(9Z))), 1-octadecanoyl-sn-glycero-3-phos­phocholine (LPC(18:0)), 1-(1Z-octadecenyl)-2-do­cosahexaenoyl-sn-glycero-3-phosphocholine (PC(18:0p/22:6)) purchased from Sigma-Al­drich.1-stearoyl-2-linoleoyl-sn-glycerol (DG(18:0/20:4)) purchased from Avanti Polar Lipids, Inc. (Alabaster, AL, USA) and tripalmitin-1,1,1-13C3 (TG(16:0/16:0/16:0)-13C3), trioctanoin-1,1,1-13C3 (TG(8:0/8:0/8:0)-13C3) and 1-palmitoyl-2-hydroxy-sn-Glycero-3phosphatidylcholine (LPC(16:0)) from La­rodan AB (Solna, Sweden).

**Supplementary Methods 2 Data analysis plan for the linear mixed effects model.**

# **Supplementary Figures**

**
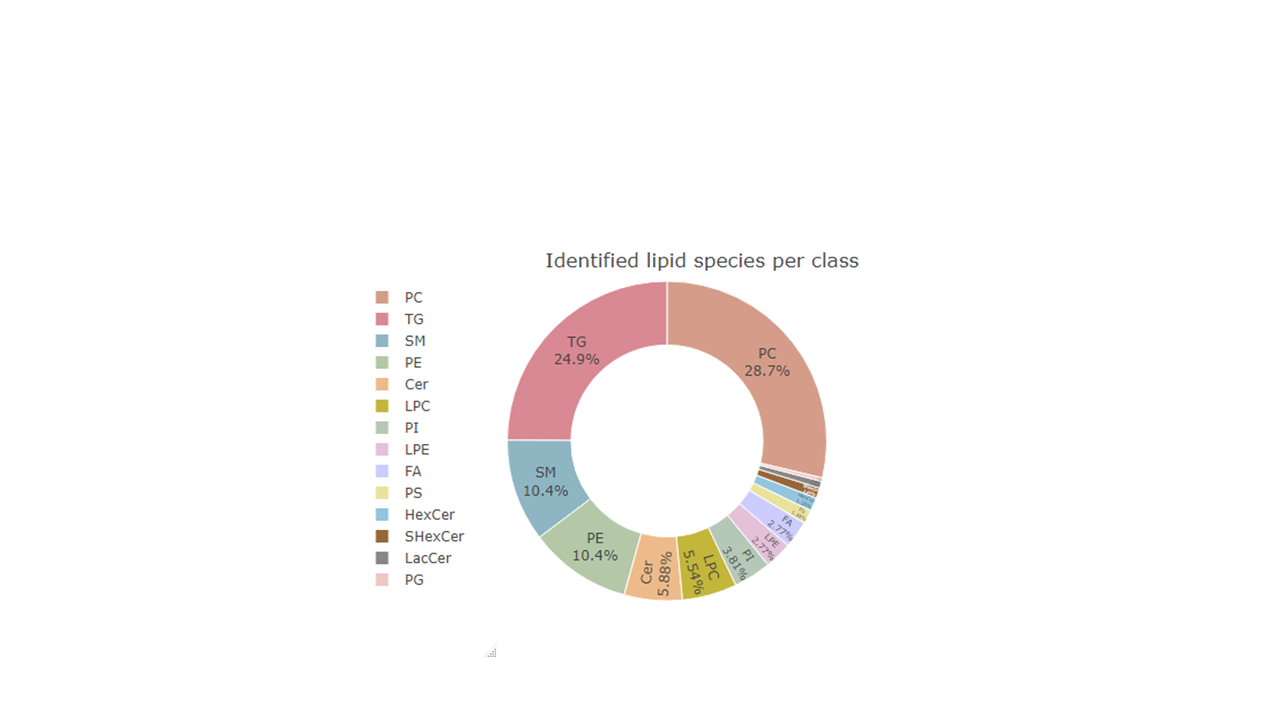
**

**Supplementary Fig. 1 The dominance of identified lipid species and their proportion (%) within their respective lipid classes.** Lipidomics data were dominated by PCs (n=83) and TGs (n=72), followed by SMs (n=30) and PEs (30). Cers, LPCs, PIs, LPEs, FFA, PSs, HexCers, SHexCers, LacCers and PGs were all annotated in a smaller amounts (n=17, 16, 11, 8,8,4,4,3,2 and 1).

# **Supplementary Tables**

**Supplementary Table 1 Technical variation in the analysis.** Shown in the table are the coefficient of variation (or, relative standard deviation; RSD; %) for peak area of the internal standards (name) in fasting plasma samples.

| Name | %RSD: peak area |
| --- | --- |
| 01. PE(17:0/17:0) | 23,34646 |
| 02. SM(d18:1/17:0) | 14,10878 |
| 03. Cer(d18:1/17:0) | 14,92515 |
| 04. PC(17:0/17:0) | 13,2443 |
| 05. LPC(17:0) | 14,14068 |
| 06. PC(14:0/d13) | 14,60675 |
| 07. TG(16:0/16:0/16:0)-13C3 | 14,656 |
| 08. TG(8:0/8:0/8:0)-13C3 | 15,16932 |
| 09. PC(16:0/d31/18:1) | 24,88088 |
| 09b. PC(16:0/d30/18:1) | 25,06463 |
| AVERAGE | **17,4143** |

**Supplementary Table 2 Results of the mixed-effects model.** Shown in the table are (Name) of individual lipid species, HDL-cholesterol slope (Slope), its lower and upper confidence intervals (L95, U95), standard error, and p-value of the slope (FDR) after correction for multiple testing.

| Name | Slope | L95 | U95 | SE | FDR |
| --- | --- | --- | --- | --- | --- |
| PC(P-36:4)/PC(O-36:5) | 1.60 | 1.02 | 2.18 | 0.28 | 2.36 x10^-5^ |
| PC(35:4) | 1.25 | 0.58 | 1.92 | 0.33 | 3.06 x10^-3^ |
| SM(d34:2) | 1.17 | 0.49 | 1.86 | 0.34 | 4.86 x10^-3^ |
| PC(P-38:4)/PC(O-38:5) | 1.09 | 0.40 | 1.79 | 0.34 | 8.32 x10^-3^ |
| SM(d36:2) | 0.99 | 0.28 | 1.70 | 0.35 | 1.84 x10^-2^ |
